# Factors Related to Prevalence of Hallux Valgus in Female University Students: A Cross-Sectional Study

**DOI:** 10.2188/jea.JE20130110

**Published:** 2014-05-05

**Authors:** Hiroto Okuda, Sachiko Juman, Ai Ueda, Tomohiro Miki, Masayuki Shima

**Affiliations:** 1 School of Pharmaceutical Sciences, Mukogawa Women’s University, Nishinomiya, Hyogo, Japan; 1 武庫川女子大学薬学部病態生理学研究室; 2 Department of Public Health, Hyogo College of Medicine, Nishinomiya, Hyogo, Japan; 2 兵庫医科大学公衆衛生学教室

**Keywords:** hallux valgus, female university student, big toe pain, family history, cross-sectional survey

## Abstract

**Background:**

We investigated the prevalence of hallux valgus (HV) and examined its association with various factors in a cross-sectional study of Japanese female university students.

**Methods:**

A questionnaire survey of foot symptoms, lifestyle, and body mass index (BMI) was administered to 343 women who provided informed consent at a women’s university. Footprints were obtained and bone density was measured. Associations of HV with various factors were analyzed by logistic regression analysis.

**Results:**

Big toe pain was reported in 26.5% of the women. HV (HV angle, ≥15°) was present in the left foot in 22.4%, the right foot in 20.7%, and unilaterally or bilaterally in 29.7% of women. Mild HV (HV angle, ≥15° to <20°) was noted in the left foot and right foot in 13.4% and 13.1% of women, respectively; no severe HV (HV angle, ≥40°) was observed. HV was associated with big toe pain (adjusted OR: 3.56, 95% CI: 2.01–6.32), history of HV in the mother or maternal grandmother (adjusted OR: 2.45, 95% CI: 1.19–5.02), and history of HV in other family members (adjusted OR: 3.09, 95% CI: 1.35–7.06). Moderate HV was associated with big toe pain (adjusted OR: 4.58, 95% CI: 2.17–9.66) and history of HV in the mother or maternal grandmother (adjusted OR: 3.36, 95% CI: 1.40–8.07). The proportion of women with big toe pain increased significantly with HV severity.

**Conclusions:**

HV was present in about 30% of female university students. Young women with big toe pain or a family history of HV should be evaluated for HV.

## Abstract (ja)

**【背景】:**

外反母趾は女性に多くみられる疾患であるが、一般の若年女性を対象とした報告は比較的少ない。そこで女子大学生における外反母趾の有病率及びそれに関連する因子を明らかにするために横断調査を実施した。

**【方法】:**

インフォームド・コンセントが得られた女子大学生343名を対象に足型を測定し、外反母趾の有無と重症度を評価した。足の症状、日常生活習慣、BMIに関するアンケート調査、骨密度測定を行い、外反母趾と各種因子との関連についてロジスティック回帰分析を用いて検討した。

**【結果】:**

26.5％の学生が母趾痛を自覚していた。外反母趾(母趾角15度以上)は左22.4％、右20.7％、1足ないし両足29.7％に認めた。そのうち軽度(母趾角15度以上20度未満)は左13.4％、右13.1％で、重度(母趾角40度以上)はいなかった。1足ないし両足の外反母趾では母趾痛有(調整オッズ比（OR）：3.56；95％信頼区間（CI）： 2.01-6.32)、母又は母方祖母に家族歴有(調整OR：2.45；95％ CI：1.19-5.02)、その他の家族歴有(調整OR：3.09；95％ CI：1.35-7.06)との関連が有意であった。中等度の外反母趾に限れば母趾痛有(調整OR：4.58；95％ CI：2.17-9.66)、母又は母方祖母に家族歴有(調整OR：3.36；95％ CI：1.40-8.07)との関連を認めた。外反母趾の重症度が上がると、母趾痛を訴える学生の割合が有意に増加した。

**【結論】:**

約3割の女子大学生に１足ないし両足の外反母趾を認めた。外反母趾の家族歴や母趾痛のある若年女性は外反母趾の可能性があり注意が必要である。

## INTRODUCTION

Hallux valgus (HV) is a deformity characterized by lateral deviation of the big toe at the first metatarsophalangeal joint and is more frequent among women.^1^ It is the most common foot deformity in adults, and its prevalence increases with age.^2^^–^^6^^,^^9^^,^^12^ In HV the first metatarsophalangeal joint protrudes due to big toe deformity, and inflammation with reddening and swelling may be induced by external stimulation from shoes. Pain and numbness of the big toe may be caused by compression of adjacent nerves. In the advanced stage, the big toe may sink under the second toe, causing callus or dislocation of the second toe. Marked deformation impairs weight bearing and balance, increases the risk of falling,^7^ and worsens physical performance and quality of daily life.^5^^,^^8^^,^^9^

HV was reported to be associated with gender,^2^^,^^4^^,^^5^^,^^9^^–^^11^ age,^2^^–^^6^^,^^9^ big toe pain,^3^^,^^5^^,^^9^^,^^12^ family history,^1^^,^^11^^,^^13^^,^^14^ footwear,^1^^,^^4^^,^^10^^,^^11^^,^^15^^–^^19^ body mass index (BMI),^9^^,^^11^ first metatarsal length,^1^^,^^10^^,^^20^^,^^21^ first metatarsal head shape,^21^ flatfoot,^10^^,^^11^^,^^22^ race,^11^^,^^23^ knee pain,^3^^,^^5^ osteoarthritis,^3^^,^^24^ and ligamentous laxity.^25^ The recent increase in HV prevalence is thought to be largely due to the effect of footwear, including high-heeled shoes, particularly in middle-aged and older people.^4^^,^^15^^–^^18^

The presence and severity of HV are evaluated on the basis of HV angle, which is usually measured clinically using radiographs. A diagnosis of HV deformity usually requires an HV angle greater than 15°.^1^ However, for ethical reasons HV severity is often evaluated in epidemiologic studies by measuring the HV angle in a traced outline of the foot rather than by using radiographs.^4^^,^^6^^,^^19^^,^^26^

Many studies have examined associations of HV with various factors in patients, general populations, and middle-aged to older people^1^^,^^3^^–^^11^^,^^13^^,^^18^^,^^20^^–^^22^^,^^24^; however, relatively few studies have investigated HV in young women.^12^^,^^26^ Reports indicate that the frequency of HV is increasing among junior high school students^27^ and that big toe deformity began before age 20 years in 46% of adults who underwent surgical treatment for HV.^13^ Thus, attention to HV and measures to prevent it at a young age are important. Opportunities to wear high-heeled footwear markedly increase as women enter university. Since such footwear greatly stresses the big toe, it likely increases the incidence and severity of HV. Young women need to be more aware of the characteristics and severity of HV and its relationship to footwear.

To assess HV prevalence, we analyzed the footprints of first-year female university students. In addition, associations of HV with various factors were investigated to identify measures to prevent HV.

## METHODS

### Participants

The participants were 634 students newly admitted in 2010–2012 to the Faculty of Pharmacy of a private women’s university in Hyogo Prefecture. Most were younger than 20 years. The survey objectives and methods were explained orally and in writing, after which the questionnaire survey forms were distributed.

Among a total of 634 first-year students admitted during the 3-year period (197 in 2010, 239 in 2011, and 198 in 2012), written consent to participate in the study was obtained from 353 (55.7%) women, and 343 women aged 20 years or younger (54.1%) were enrolled. A total of 258 (75.2%) participants lived with their families and commuted to and from the university. The survey was carried out on December 7–10, 2010; November 30 to December 3, 2011; and December 11–15, 2012.

### Questionnaire survey on foot symptoms and lifestyle

A self-administered questionnaire contained items on age, body weight, height, and hallux valgus. In particular, big toe pain was assessed according to frequency of pain (none, occasional, always), time of pain (during rest/night, during walking/exercising, others), and treatment status for pain. The participants were also asked whether they had experienced knee pain and foot fatigue. Family history of HV was defined as presence of a relative (within the second degree of consanguinity) believed to have HV, and participants were asked to identify the relationship of such relatives with the participant. Footwear was assessed according to whether the participant used high heels with a narrow tow box, heel height, frequency of high heel use (none, occasional, every day), and duration of high heel use. Finally, participants were asked, in an open question, about sports they had played and how long they had played them.

### Footprint measurement

In footprints obtained from plantar images, the HV angle was measured, the presence and severity of HV were evaluated, and the lengths of first and second toes were compared. Plantar images were obtained using Foot Look (FOOTLOOK Inc., Fukuoka, Japan), a device for plantar balance measurement that has a scanner capable of high-speed scanning of the plantar surface of the foot. The participants stood on the instrument barefooted, and the plantar surfaces in a weight-bearing state were scanned 3 times. The scanned images were printed on A4 paper, and HV angle was measured using outlines of full-scale footprints printed on paper.

**Figure 1.**
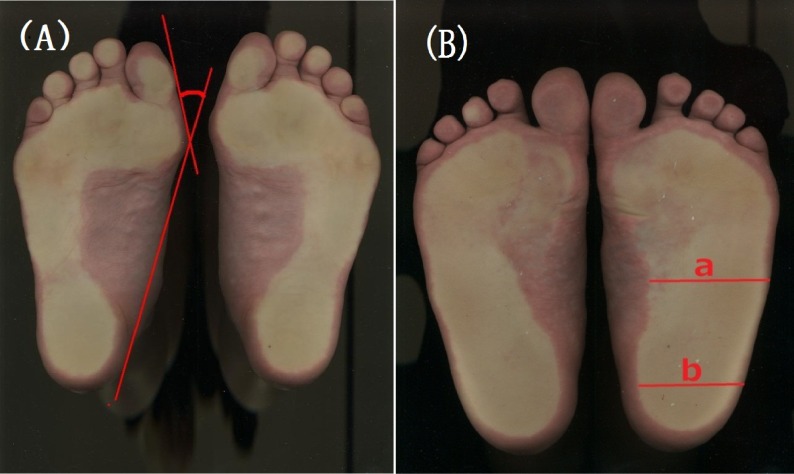
Use of footprints to measure (A) HV angle and (B) flatfoot. (A) A straight line is drawn through the most medial points of the first metatarsophalangeal joint and the heel (inside edge). A second straight line is drawn through the first metatarsophalangeal joint and the great toe (proximal phalanx). The hallux valgus angle is defined as the angle between these 2 straight lines. (B) Measurement of arch width in the central region (a) and heel region (b) of the foot. The Staheli Arch Index is calculated as (a)/(b).

For ethical reasons, radiographs were not used to assess HV in this study. The presence and severity of HV were evaluated according to HV angles determined from foot outlines.^4^^,^^6^^,^^19^^,^^26^ As shown in Figure 1(A), HV angle was measured as the angle between the tangent of the big toe and the medial tangent of the heel at the varus protrusion of the first metatarsophalangeal joint in the contour of the footprint. HV angle measurements based on foot outlines and radiographs were found to be highly correlated (*r* = 0.955, *P* < 0.0001).^6^ Thus, the present technique of footprint measurement has been validated.^4^^,^^6^^,^^19^

Regarding assessment of severity, HV angles of 15° to 19°, 20° to 39°, and 40° or greater were classified as mild, moderate, and severe, respectively, according to the criteria on angular measurements of the ad hoc committee of the American Orthopedic Foot and Ankle Society.^28^

Flatfoot was judged using the Staheli Arch Index (SAI), which is significantly correlated with 3-dimensional arch structure on radiographs. As shown in Figure 1(B), flatfoot was defined as a central-to-heel arch width ratio of the plantar surface in the footprint (SAI) of 1.0 or greater.^29^

The minimum values for HV angle and SAI in the 3 scans were used in the analysis. The lengths of the first and second toes were measured along the long axes of the toes, from the most recessed point between the 2 toes in the plantar image.

All measurements in the present study were done by the same physician, who has been engaged in foot care and treatment for more than 15 years.

### Measurement of bone density

The bone density of the right calcaneus was measured using an ultrasound bone densitometer (AOS-100, ALOKA, Ltd., Tokyo, Japan). The osteosono-assessment index (OSI), which reflects bone density and bone mass, was calculated from the speed of sound passing through the right calcaneus, and the transmission index indicates the degree of ultrasound transmission. Bone mass was defined as reduced when the OSI was less than 2.428 (YAM-0.9SD; YAM: young adult mean, SD: standard deviation).^30^

### Statistical analysis

Various factors were examined, including those associated with HV in previous studies.^1^^–^^6^^,^^8^^–^^25^ The relationship between mean HV angle and HV frequency was evaluated in relation to big toe pain (always, occasional, none), knee pain (present, absent), year of university admission, foot fatigue (present, absent), family history of HV (present, other), high heels with a narrow toe box (≥6 cm, <6 cm, do not wear), high heels 6 cm or higher (wear every day, wear occasionally, do not wear), big toe length (longer than the second toe, not longer than the second toe), flatfoot (present, absent), history of sports for 6 years or longer (ballet, sports other than ballet, none), BMI (<19.0 kg/m^2^, 19.0–21.4 kg/m^2^, ≥21.5 kg/m^2^), and OSI (≥2.428, <2.428). Differences in mean values were examined by the *t* test, differences in ratios by the χ^2^ test, and multiple comparisons were done using the Tukey procedure. To examine the association between HV severity and big toe pain, BMI- and age-adjusted logistic regression analysis was used to estimate adjusted odds ratios (ORs) and 95% confidence intervals (CIs). Logistic regression analyses were used to compare factors (big toe pain, knee pain, year of university admission, family history of HV, use of high heels with a narrow toe box, big toe length, flatfoot, sports history, BMI, and OSI) between women with and without HV, and adjusted odds ratios and 95% CIs were estimated. The analyses were performed using SPSS 19.0J for Windows. Statistical significance was defined as a *P* value less than 0.05 in all tests.

### Ethical considerations

The survey was conducted with the approval of the Research Review Board of Mukogawa Women’s University. The participants were enrolled in the survey only after they had given their written consent after receiving an explanation of the study objectives and methods using explanation/recruitment and consent forms.

## RESULTS

### Characteristics of participants

Table 1 shows the number of women by year, enrollment rate, mean age, height, body weight, BMI, OSI, and percentages with big toe pain, knee pain, and foot fatigue. About 26.5% of participants complained of big toe pain, but only 4.3% had a history of treatment for big toe pain. A family history of HV was noted in 25.4%. The Japanese translation of the term “hallux valgus” was known to 92.6% of participants.

**Table 1.** Demographic characteristic of participants (*n* = 343)

Age in years, mean (SD)	18.7 (0.6)
Weight in kg, mean (SD)	51.2 (7.6)
Height in cm, mean (SD)	157.8 (5.4)
BMI in kg/m^2^, mean (SD)	20.6 (2.7)
OSI, mean (SD)	2.704 (0.276)

Year of admission, fiscal year	
2010, no. (%)	131 (38.2)
2011, no. (%)	105 (30.6)
2012, no. (%)	107 (31.2)

Knee pain, no. (%)	23 (6.7)
Foot fatigue, no. (%)	114 (33.2)
Big toe pain, no. (%)	91 (26.5)
Period with big toe pain	
During walking/exercising, no. (%)	79 (23.0)
During rest/nighttime, no. (%)	8 (2.3)
Others, no. (%)	4 (1.2)
Frequency of big toe pain	
Occasional, no. (%)	84 (24.5)
Always, no. (%)	7 (2.0)

Family history, no. (%)	86 (25.4)
Mother, no. (%)	39 (11.4)
Father, no. (%)	8 (2.3)
Sibling, no. (%)	20 (5.8)
Maternal grandmother, no. (%)	17 (5.0)
Paternal grandmother, no. (%)	24 (4.1)
Grandfather, no. (%)	4 (1.2)

BMI: body mass index; OSI: osteosono-assessment index.

### HV angle and severity

**Figure 2.**
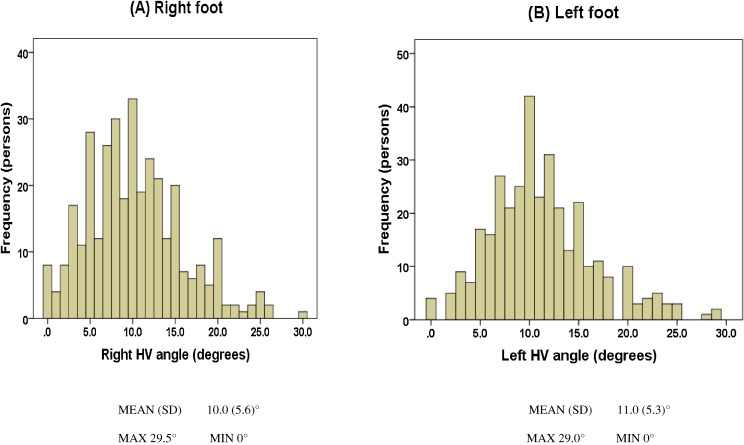
Frequency distribution of HV angle. Correlation of the HV angles of the left and right feet (Pearson correlation coefficient: 0.711, *P* < 0.001).

Figure 2 shows the frequency distribution of HV angles in both feet. The mean angle was 10.0° for the right foot and 11.0° for the left foot, and the 2 sides were strongly correlated (Pearson correlation coefficient: 0.711, *P* < 0.001). Table 2 shows the frequencies of HV by severity. No severe HV was observed. About 29.7% of women had HV in at least 1 foot.

**Table 2.** Proportions of participants with HV in the left and right feet, by severity

		HV angle of right foot		

		Normal (<15°)	Mild (15–19°)	Moderate (≥20°)
HV angle of left foot	Normal (<15°)	241 (70.3%)	19 (5.5%)	6 (1.7%)
	Mild (15–19°)	21 (6.1%)	18 (5.2%)	7 (2.0%)
	Moderate (≥20°)	10 (2.9%)	8 (2.3%)	13 (3.8%)

				No. (%)

### Associations of mean HV angle and HV frequency with the investigated factors

Table 3 shows the mean HV angles of the right foot and left foot in relation to the examined factors. The mean HV angles of both feet were significantly larger among women with a higher prevalence of big toe pain and a family history of HV in the mother or maternal grandmother. In the right foot, the mean HV angle was significantly larger among women with a big toe longer than the second toe than among those with a big toe not longer than the second toe.

**Table 3.** Mean HV angles of the left and right feet in relation to investigated factors

	Factor	Right foot			Left foot		
	
		No.	Mean degrees (SD)	*P* value	No.	Mean degrees (SD)	*P* value
Big toe pain	Absent	252	9.1 (5.0)	<0.001	252	10.1 (4.6)	<0.001
	Occasional	84	12.1 (6.1)		84	13.2 (5.9)	
	Always	7	18.0 (8.3)		7	19.1 (6.8)	
Knee pain	Absent	320	10.1 (5.8)	0.729	320	11.1 (5.3)	0.531
	Present	23	9.7 (5.9)		23	10.3 (5.5)	
Foot fatigue	Absent	229	10.0 (5.4)	0.811	229	11.0 (5.3)	0.945
	Present	114	10.1 (6.0)		114	11.0 (5.4)	
Family history	Absent	257	9.3 (5.3)	<0.001	257	10.4 (5.0)	0.001
	Mother/maternal grandmother	51	12.3 (6.2)^a^		51	13.2 (6.0)^a^	
	Other than mother/maternal grandmother	35	12.3 (5.5)		35	12.2 (5.1)	
High heels with a narrow toe box	Absent	124	9.9 (5.8)	0.977	124	10.9 (5.4)	0.775
	<6 cm	151	9.9 (5.4)		151	10.8 (5.1)	
	≥6 cm	51	10.1 (5.4)		51	11.4 (5.7)	
Frequency of the use of high heels with a narrow toe box (≥6 cm)	Absent	276	9.9 (5.7)	0.990	276	10.9 (5.4)	0.824
	Occasional	25	10.1 (4.6)		25	11.5 (6.1)	
	Every day	26	10.1 (6.1)		26	11.3 (5.3)	
Big toe length	≤second toe length	66	8.6 (5.0)	0.022	57	10.4 (5.5)	0.374
	>second toe length	277	10.4 (5.7)		286	11.1 (5.2)	
Flatfoot	Absent	332	10.0 (5.5)	0.100	333	11.0 (5.3)	0.454
	Present	11	12.8 (8.3)		10	12.3 (6.0)	
Athletic history (≥6 years)	Absent	200	9.9 (5.5)	0.360	200	11.0 (5.3)	0.987
	Ballet	12	8.5 (4.5)		12	10.9 (3.8)	
	Other than ballet	124	10.5 (5.0)		124	11.0 (5.3)	
BMI	<19.0 kg/m^2^	80	10.3 (5.8)	0.846	80	11.4 (5.3)	0.711
	19.0–21.4 kg/m^2^	175	9.9 (5.5)		175	10.9 (5.2)	
	≥21.5 kg/m^2^	86	10.1 (5.7)		86	10.8 (5.5)	
OSI	≥2.428	295	9.8 (5.5)	0.059	295	10.9 (5.1)	0.591
	<2.428	45	11.4 (5.9)		45	11.4 (6.1)	

^a^Mean HV angle was significantly larger than in the group with no family history (*P* = 0.001).

BMI: body mass index; OSI: osteosono-assessment index.

Table 4 and eTable 1 show the frequencies of HV in relation to the investigated factors. HV frequency was significantly higher among women with a higher frequency of big toe pain and among those with a family history of HV.

**Table 4.** Prevalence of HV (HV angle ≥15°) in relation to investigated factors

Factor		Right foot			Left foot			At least 1 foot		
		
		No.	%	*P* value	No.	%	*P* value	No.	%	*P* value
Big toe pain frequency	Absent	252	14.3	<0.001	252	16.7	<0.001	252	22.2	<0.001
	Occasional	84	35.7		84	35.7		84	48.8	
	Always	7	71.4		7	71.4		7	71.4	
Knee pain	Absent	320	20.3	0.593	320	22.2	0.613	320	29.1	0.346
	Present	23	26.1		23	26.1		23	39.1	
Year of admission	2010	131	25.2	0.262	131	25.2	0.585	131	32.8	0.535
	2011	105	17.1		105	21.9		105	29.5	
	2012	107	18.7		107	19.6		107	26.2	
Foot fatigue	Absent	229	19.2	0.396	229	23.1	0.683	229	29.3	0.803
	Present	114	23.7		114	21.1		114	30.7	
Family history	Absent	257	17.1	0.016	257	17.1	<0.001	257	23.7	<0.001
	Mother/ maternal grandmother	51	33.3		51	49.0		51	49.0	
	Other than the mother/ maternal grandmother	35	28.6		35	45.7		35	45.7	
High heels with a narrow toe box	Absent	124	20.1	0.430	124	21.8	0.759	124	27.4	0.358
	<6 cm	151	17.2		151	20.5		151	27.2	
	≥6 cm	51	25.5		51	25.5		51	37.3	
Frequency of the use of high heels with a narrow toe box	Absent	276	18.8	0.532	276	21.4	0.739	276	27.5	0.341
	Occasional	25	20.0		25	28.0		25	40.0	
	Every day	26	26.9		26	23.1		26	34.6	
Big toe length	≤second toe length	66	13.6	0.130	57	21.1	0.863	—	—	—
	>second toe length	277	22.4		286	22.7		—	—	—
Flatfoot	Absent	332	20.2	0.248	333	22.2	0.699	328	29.3	0.393
	Present	11	36.4		10	30.0		15	40.0	
Athletic history ≥6 years	Absent	200	22.0	0.513	200	24.0	0.644	200	30.5	0.899
	Ballet	12	8.3		12	16.7		12	25.0	
	Other than ballet	124	20.2		124	20.2		124	29.0	
BMI	<19.0 kg/m^2^	80	21.3	0.962	80	21.3	0.812	80	31.3	0.941
	19.0–21.4 kg/m^2^	175	21.1		175	24.0		175	29.1	
	≥21.5 kg/m^2^	86	19.8		86	20.9		86	30.2	
OSI	≥2.428	295	20.2	0.695	295	21.4	0.442	295	28.5	0.487
	<2.428	45	22.2		45	26.7		45	33.3	

BMI: body mass index; OSI: osteosono-assessment index.

### Results of logistic regression analyses

Table 5 shows data on the association between HV and big toe pain. The frequency of big toe pain increased significantly in relation to HV severity, after adjustment for BMI and age.

**Table 5.** Frequency of big toe pain according to severity

	No.	%	Odds ratio^a^ (95% CI)	*P* value
HV angle of right foot				
Normal	272	20.6	1.00	
Mild	45	42.2	2.86 (1.47–5.57)	0.002
Moderate	26	65.4	7.25 (3.03–17.34)	<0.001

HV angle of left foot				
Normal	266	21.1	1.00	
Mild	46	37.0	2.21 (1.13–4.34)	0.021
Moderate	31	63.2	5.93 (2.69–13.07)	<0.001

^a^Adjusted for age and body mass index by logistic regression.

Table 6 and eTable 2 show the results of logistic regression analyses of the investigated factors. In our participants, the presence of HV (HV angle, ≥15°) in at least 1 foot was related to presence of big toe pain, family history of HV in the mother or maternal grandmother, and family history in other relatives. In addition, the presence of moderate HV in at least 1 foot was related to presence of big toe pain and family history of HV in the mother or maternal grandmother.

**Table 6.** Odds ratio (ORs) and 95% CIs for an HV angle ≥15° in relation to investigated factors

Factor		Right foot		Left foot		At least 1 foot	
		
		Odds ratio^a^ (95% CI)	*P* value	Odds ratio^a^ (95% CI)	*P* value	Odds ratio^a^ (95% CI)	*P* value
Big toe pain frequency	Absent	1.00		1.00		1.00	
	Occasional	3.01 (1.56–5.81)	0.001	2.29 (1.21–4.31)	0.011	3.56 (2.01–6.32)	<0.001
	Always	21.52 (2.15–215.30)	0.009	23.48 (2.38–231.67)	0.007		
Knee pain	Absent	1.00		1.00		1.00	
	Present	1.32 (0.40–4.34)	0.650	0.90 (0.26–3.16)	0.874	1.46 (0.50–4.29)	0.489
Year of admission	2010	1.00		1.00		1.00	
	2011	0.66 (0.31–1.43)	0.290	0.80 (0.39–1.61)	0.528	0.82 (0.42–1.59)	0.554
	2012	0.79 (0.38–1.64)	0.526	0.79 (0.38–1.61)	0.510	0.91 (0.48–1.72)	0.761
Family history	Absent	1.00		1.00		1.00	
	Mother/ maternal grandmother	1.94 (0.86–4.37)	0.110	2.78 (1.31–5.91)	0.008	2.45 (1.19–5.02)	0.007
	Other than the mother/ maternal grandmother	1.85 (0.72–4.74)	0.199	3.63 (1.56–8.49)	0.003	3.09 (1.35–7.06)	0.007
High heels with a narrow toe box	Absent	1.00		1.00		1.00	
	<6 cm	0.75 (0.39–1.47)	0.403	0.85 (0.44–1.61)	0.610	0.89 (0.49–1.61)	0.705
	≥6 cm	1.46 (0.63–3.40)	0.380	1.27 (0.55–2.95)	0.573	1.68 (0.78–3.62)	0.187
Big toe length	≤second toe length	1.00		1.00		—	—
	>second toe length	1.61 (0.68–3.81)	0.278	0.80 (0.37–1.73)	0.566	—	—
Flatfoot	Absent	1.00		1.00		1.00	
	Present	1.57 (0.37–6.79)	0.543	1.56 (0.33–7.29)	0.575	1.65 (0.50–5.46)	0.410
Athletic history	<6 years	1.00		1.00		1.00	
	≥6 years	0.89 (0.65–1.22)	0.462	0.93 (0.69–1.26)	0.630	0.92 (0.70–1.21)	0.535
BMI	<21.5 kg/m^2^	1.00		1.00		1.00	
	≥21.5 kg/m^2^	1.05 (0.51–2.16)	0.906	1.14 (0.58–2.25)	0.718	1.24 (0.66–2.31)	0.508
OSI	≥2.428	1.00		1.00		1.00	
	<2.428	1.43 (0.62–3.29)	0.398	1.60 (0.72–3.52)	0.246	1.48 (0.70–3.11)	0.307

^a^Adjusted for all variables in table by logistic regression.

## DISCUSSION

### HV prevalence

In this study, HV, as determined using the HV angle measured in footprints, was observed in at least 1 foot of 29.7% of female first-year university students aged 20 years or younger. Moderate HV was noted in 12.8%. In a meta-analysis of 78 reports of HV primarily in the United States and United Kingdom the prevalence of HV was 15.0% (95% CI: 7.7%–22.3%) among people younger than 18 years and 26.3% (95% CI: 16.5–36.2) among those aged 18 to 65 years.^2^ In a 2004 study by Shibata et al, which used the trace method to study Japanese women, the mean HV angle was 10.0° among those aged 10 to 19 years and 13.0° among those aged 20 to 29 years; the prevalence of moderate HV (HV angle, ≥20°) was 12.5% among those aged 20 to 29 years.^6^ Mean HV angle and HV prevalence in the present study were very similar to values from previous reports.

### HV angle and severity

HV is diagnosed according to HV angle, which is usually measured clinically using radiographs.^1^ In epidemiologic studies of healthy people, HV severity is often evaluated by applying criteria such as the Manchester scale to photographs or diagrams of the foot^3^^,^^5^^,^^10^^,^^12^^,^^31^^,^^32^ or by measuring the HV angle in the outlines of foot traces.^4^^,^^6^^,^^19^^,^^26^ In this study, HV angle was measured using the outlines of plantar images obtained with a scanner, because this technique is known to yield values similar to those obtained using radiographs.^4^^,^^6^^,^^19^^,^^33^ This method is advantageous because it does not require X-ray exposure, is noninvasive, allows for more-objective evaluation of the plantar surface, easier data management, and superior device portability, and is considered appropriate for surveying a large number of participants.

### HV and related factors

HV is usually a bilateral phenomenon—there is typically little difference between the right and left foot.^3^^,^^12^ However, since there were differences between the left and right feet in the length of the big toe and prevalence of flatfoot, the relationships between HV and various factors were examined for each and both feet. The results showed similar associations between HV and the investigated factors.

#### Relationship with big toe pain

Big toe pain was reported by 26.5% of the participants during walking or exercising, if not regularly. A number of studies examined the relationship between the presence of HV and big toe pain^3^^,^^5^^,^^8^^,^^9^^,^^12^; some found no association,^11^ but the question remains open. The severity of big toe pain is believed to depend on the method used to assess pain, the characteristics and ethnicity of the study population, shoes currently worn, and history of treatment for HV.^3^^,^^9^^,^^11^ The present results showed a significant association between HV and big toe pain, presumably because the participants were young women and big toe pain was partly caused by intense exercise and walking, which imposed much strain on their feet. Young women tend to be more active than middle-aged and elderly women and often wear fashion-oriented footwear, including high-heeled shoes, which puts direct pressure on the first metatarsophalangeal joint. Furthermore, they have few opportunities to receive treatment for HV and advice on appropriate footwear, even if they have HV.

In this study, HV prevalence was significantly higher in women with big toe pain as compared with those without such pain. In addition, the percentage of participants with big toe pain increased significantly with HV severity. However, because most participants had HV without big toe pain, attention to asymptomatic HV is necessary.

#### Associations with causative factors

Internal factors such as anatomic characteristics associated with genetic predispositions and external factors such as footwear may be involved in the development of HV.^1^ In this study, HV prevalence was significantly higher in women with a family history of HV than in those without such a history.

Previous studies reported that 63% to 84% of patients scheduled to undergo surgical correction of HV had a family history of HV and that maternal inheritance was present in 68% to 94% of those with such a history.^1^^,^^10^^,^^13^ Moreover, in a study of French patients with painful HV, more than 90% had a history extending over 3 generations, which suggests the presence of incomplete autosomal dominant inheritance.^14^ The high frequency of family occurrence suggests the involvement of inheritance of anatomic and morphologic characteristics that increase vulnerability to HV, such as foot morphology, joint shape, and ligament flexibility.

In this study, the prevalence of moderate HV was significantly higher in participants with a mother or maternal grandmother with HV than in those without such a family history, even after adjustment for other factors, which suggests maternal inheritance. Among all participants with HV, 47.7% had a family history of HV, and maternal inheritance was suspected in 60.4% of those with a family history. These proportions are lower than those reported previously, probably because the populations differed in age and HV severity.

Regarding foot morphology, the mean HV angle of the right foot was significantly larger in women with a big toe longer than the second toe, but no significant difference was noted in HV prevalence. Also, the presence of flatfoot was not related to mean HV angle or HV prevalence. Generally, feet with a big toe longer than the second toe (Egyptian type) are more susceptible to the effects of footwear and weight-bearing and are more likely to develop HV than feet with a big toe shorter than or equal in length to the second toe (Greek type; square type).^21^ However, the effects of big toe length are considered to be limited in young people.

External factors related to HV include footwear^1^^,^^4^^,^^10^^,^^11^^,^^15^^–^^19^ and overweight.^9^^,^^34^ The human foot has 3 arches, which support body weight, efficiently transmit power to the ground during locomotion, and absorb impact during walking. Loss of these arch functions due to external factors leads to HV. When shoes with a narrow toe box are worn, the big toe, which is normally introverted by contraction of the abductor muscle during weight bearing, is compressed in the valgus position. Moreover, as shoes with higher heels are worn, the foot is fixed in a more dorsiflexed position, and a stronger force is applied to the anterior foot. This flattens the transverse arch and makes the foot more vulnerable to HV. Shine et al studied HV prevalence among residents of the island of Saint Helena and reported that HV was present in 2% or fewer of those who did not wear shoes in daily life, as compared with 16% of men and 48% of women who had worn shoes in daily life for 60 years or longer.^4^ HV was also reported to be prevalent in ballet dancers, who wear narrow toe shoes and dance by tiptoeing.^1^ However, among the present participants, no significant association was noted between HV and external factors such as fashionable footwear, ballet experience, and high BMI. This absence of a relationship may be explained by limited use of high heels, the low percentage of participants practicing ballet, and the short time spent practicing ballet as compared with professional ballet dancers. In addition, the self-reported BMI (20.6 ± 2.7 kg/m^2^) of participants was similar to the mean BMI of all newly admitted students over the past 3 years (20.9 ± 3.1 kg/m^2^), which was calculated based on data obtained at check-ups. Most participants had a lower BMI than that of middle-aged and elderly people, as reported in a previous study,^9^ few participants were obese, and the effects of BMI on the feet were likely minor. Thus, genetic factors are considered to have an important role in HV in young women, and the effects of external factors such as footwear appear to be limited. However, in children younger than school age, HV angle was reported to increase when shoe length was short for the foot,^19^ so the effects of the duration of use and size of footwear other than high heels need to be evaluated further.

### Study limitations

First, consent for participation in the study was obtained from only 55.7% of all students. Reasons for refusal to consent are believed to include aversion to having others observe their plantar images and unwillingness to participate; however, to avoid bias it is important to improve the consent rate in future studies. Second, this study was cross-sectional, and longitudinal follow-up of changes in HV angle is necessary in order to evaluate associations of onset and exacerbation with external factors. Moreover, evaluation of the effects of footwear other than toe shoes and high heels is necessary. Third, footprints were used to evaluate HV. The use of radiographs as a reference would have allowed more-accurate measurement of HV angle, ascertainment of flatfoot, comparison of big toe and second toe lengths, and evaluation of the relationship of HV with the metatarsal head, osteoarthritis, etc. Fourth, since HV can be induced by diseases that cause ligamentous laxity such as Ehlers-Danlos syndrome, examination for underlying diseases is also necessary. Fifth, because family history of HV, weight, and height were evaluated using a self-administered questionnaire, the present results may be inaccurate. To perform a more accurate evaluation, it is important to assess family members in an objective manner, using their footprints, and to measure their height and body weight during footprint measurements; however, self-reported BMI (20.6 ± 2.7 kg/m^2^) was similar to the BMI (20.7 ± 2.6 kg/m^2^) of women of the same age, as indicated by data collected in the Japan National Health and Nutrition Survey in 2011.^36^

### Study significance and future research

Although HV is a common foot deformity among adult women, there have been few studies of HV in young women. In this study, we evaluated HV in a general population of young women and its associations with various factors and confirmed the association of HV with big toe pain and family history of HV in this population. Therefore, young women with big toe pain or a family history of HV should be evaluated for HV, to identify the condition at an early stage.

Young women often value style over function in footwear. Prolonged use of shoes with a narrow toe box or high heels may lead to development or exacerbation of HV. HV can be exacerbated by internal and external factors, particularly among those with a family history of HV (especially in the mother or maternal grandmother). Future longitudinal studies should attempt to clarify the effects of external factors on the development and exacerbation of HV in young women. The results of this study provide basic data that will assist in obtaining accurate assessments.

### Conclusion

HV in young women was significantly positively associated with big toe pain and family history of HV but not with external factors such as heel height or morphologic characteristics such as big toe length and flatfoot. The percentage of participants who reported big toe pain increased with HV severity. HV should be considered among young women with big toe pain or family history of HV.

## ONLINE ONLY MATERIALS

eTable 1. Incidence of HV (HV angle ≥20%) in relation to investigated factors.

eTable 2. Odds ratios (ORs) and 95% CIs for an HV angle ≥20° in relation to investigated factors.

Abstract in Japanese.
