# Supplementary material for: Factors Related to Prevalence of Hallux Valgus in Female University Students: A Cross-Sectional Study
Source: J Epidemiol. 2014 May 5;24(3):200–8. doi: 10.2188/jea.JE20130110 (PMC4000767; doi:10.2188/jea.JE20130110)
Supplement: eTable 1. [file je-24-200-s001.pdf]

eTable 1: Incidence of HV (HV angle ≥20%) in relation to investigated factors

Factor		Right foot			Left foot			At least 1 foot		
		No.	%	p value	No.	%	p value	No.	%	p value
Big toe pain Frequency	Absent	252	3.6	<0.001	252	4.8	<0.001	252	7.6	<0.001
	Occasional	84	15.5		84	19.0		84	26.2	
	Always	7	57.1		7	42.9		7	57.1	
Knee pain	Absent	320	7.5	0.690	320	9.4	0.708	320	13.4	0.751
	Present	23	8.7		23	4.3		23	8.7	
Year of admission	2010	131	7.6	0.999	131	9.9	0.593	131	13.7	0.954
	2011	105	7.6		105	6.7		105	12.4	
	2012	107	7.5		107	10.3		107	13.1	
Foot fatigue	Absent	229	6.6	0.386	229	8.7	0.842	229	11.8	0.312
	Present	114	9.6		114	9.6		114	15.8	
Family history	Absent	257	4.7	0.002	257	6.6	0.003	257	10.1	0.009
	Mother/maternal grandmother	51	17.6		51	21.6		51	25.5	
	Other than the mother/maternal grandmother	35	14.3		35	8.6		35	17.1	
High heels with a narrow toe box Height	Absent	124	5.6	0.471	124	9.7	0.942	124	12.9	0.989
	<6 cm	151	9.3		151	8.6		151	13.2	
	≥6 cm	51	5.9		51	9.8		51	13.7	
Frequency of the use of high heels with a narrow toe box	Absent	276	8 .0	0.263	276	9.4	0.908	276	13.4	0.501
	Occasional	25	0.0		25	8.0		25	8.0	
	Every day	26	11.5		26	11.5		26	19.2	
Big toe length	≤second toe length	66	6.1	0.797	57	10.5	0.618	–	–	–
	>second toe length	277	8.0		286	8.7		–	–	–
Flatfoot	Absent	332	6.9	0.040	333	8.7	0.226	328	12.5	0.119
	Present	11	27.3		10	20.0		15	26.6	
Athletic history ≥6 years	Absent	200	9.0	0.324	200	9.0	0.532	200	14.5	0.322
	Ballet	12	0.0		12	0.0		12	0.0	
	Other than ballet	124	5.6		124	9.7		124	12.1	
BMI	<19.0 kg/m^2	80	10.0		80	12.5		80	16.3	
	19.0-21.4 kg/m^2	175	6.9	0.658	175	8.0	0.479	175	12.0	0.643
	≥21.5 kg/m^2	86	7.0		86	8.1		86	12.8	
OSI	≥2.428	295	6.4	0.120	295	7.8	0.094	295	11.5	0.145
	<2.428	45	13.3		45	15.6		45	20.0	

BMI: body mass index; OSI: osteosono-assessment index.
