# Supplementary material for: Factors Related to Prevalence of Hallux Valgus in Female University Students: A Cross-Sectional Study
Source: J Epidemiol. 2014 May 5;24(3):200–8. doi: 10.2188/jea.JE20130110 (PMC4000767; doi:10.2188/jea.JE20130110)
Supplement: eTable 2. [file je-24-200-s002.pdf]

eTable 2: Odds ratios (ORs) and 95% CIs for an HV angle ≥20° in relation to various factors

Factor		Right foot		Left foot		At least 1 foot	
		**Odds ratio* (95% CI)**	**p value**	**Odds ratio* (95% CI)**	**p value**	**Odds ratio* (95% CI)**	**p value**
Big toe pain	Absent	1.00		1.00		1.00	
Frequency	Occasional	3.65 (1.28-10.48)	0.016	4.70 (1.89-11.74)	0.001	4.58 (2.17-9.66)	<0.001
	Always	31.48 (3.76-263.61)	0.001	14.05 (1.73-113.82)	0.013		
Knee pain	Absent	1.00		1.00		1.00	
	Present	0.94 (0.14-6.32)	0.945	0.19 (0.02-1.88)	0.154	0.39 (0.07-2.11)	0.272
Year of admission	2010	1.00		1.00		1.00	
	2011	1.06 (0.30-3.75)	0.933	0.32 (0.09-1.12)	0.075	0.65 (0.25-1.66)	0.367
	2012	1.26 (0.36-4.39)	0.719	0.86 (0.32-2.32)	0.770	1.02 (0.43-2.42)	0.959
Family history	Absent	1.00		1.00		1.00	
	Mother/maternal grandmother	4.54 (1.47-14.08)	0.009	4.35 (1.64-11.50)	0.003	3.36 (1.40-8.07)	0.007
	Other than the mother/maternal grandmother	2.80 (0.63-12.42)	0.174	0.92 (0.17-4.87)	0.921	1.29 (0.38-4.33)	0.685
High heels with a narrow toe box	Absent	1.00		1.00		1.00	
	<6 cm	1.47 (0.49-4.39)	0.487	0.75 (0.29-1.97)	0.564	0.89 (0.40-1.99)	0.784
	≥6 cm	0.74 (0.13-4.21)	0.736	0.87 (0.24-3.23)	0.837	0.96 (0.32-2.84)	0.935
Big toe length	≤second toe length	1.00		1.00		–	–
	>second toe length	0.84 (0.23-3.02)	0.791	0.66 (0.22-1.97)	0.457	–	–
Flatfoot	Absent	1.00		1.00		1.00	
	Present	3.42 (0.57-20.52)	0.179	2.22 (0.33-14.75)	0.409	2.27 (0.57-8.97)	0.242
Athletic history	<6 years	1.00		1.00		1.00	
	≥6 years	0.75 (0.44-1.26)	0.279	0.93 (0.59-1.44)	0.734	0.80 (0.55-1.16)	0.236
BMI	<21.5 kg/m^2	1.00		1.00		1.00	
	≥21.5 kg/m^2	0.94 (0.28-3.16)	0.918	0.92 (0.32-2.63)	0.874	0.95 (0.40-2.27)	0.905
OSI	≥2.428	1.00		1.00		1.00	
	<2.428	3.01 (0.93-9.80)	0.066	2.64 (0.91-7.65)	0.073	2.09 (0.83-5.28)	0.117

*Adjusted for all variables in table by logistic regression.
