# Supplementary material for: Factors Related to Prevalence of Hallux Valgus in Female University Students: A Cross-Sectional Study
Source: J Epidemiol. 2014 May 5;24(3):200–8. doi: 10.2188/jea.JE20130110 (PMC4000767; doi:10.2188/jea.JE20130110)
Supplement: Abstract in Japanese. [file je-24-200-s003.pdf]

女子大学生における外反母趾の有病率とそれに関連する因子:横断調査

奥田浩人[1,2]、十万佐知子[1]、植田愛[1]、三木知博[1]、島正之[2]

[1] 武庫川女子大学薬学部病態生理学研究室、[2] 兵庫医科大学公衆衛生学教室

【背景】外反母趾は女性に多くみられる疾患であるが、一般の若年女性を対象とした報告は比較的少ない。そこで女子大学生における外反母趾の有病率及びそれに関連する因子を明らかにするために横断調査を実施した。

【方法】インフォームド・コンセントが得られた女子大学生 343 名を対象に足型を測定し、外反母趾の有無と重症度を評価した。足の症状、日常生活習慣、BMI に関するアンケート調査、骨密度測定を行い、外反母趾と各種因子との関連についてロジスティック回帰分析を用いて検討した。

【結果】26.5%の学生が母趾痛を自覚していた。外反母趾(母趾角 15 度以上)は左 22.4%、右 20.7%、1 足ないし両足 29.7%に認めた。そのうち軽度(母趾角 15 度以上 20 度未満)は左 13.4%、右 13.1%で、重度(母趾角 40 度以上)はいなかった。1 足ないし両足の外反母趾では母趾痛有(調整オッズ比(OR):3.56;95%信頼区間(CI):2.01-6.32)、母又は母方祖母に家族歴有(調整 OR:2.45;95% CI:1.19-5.02)、その他の家族歴有(調整 OR:3.09;95% CI:1.35-7.06)との関連が有意であった。中等度の外反母趾に限れば母趾痛有(調整 OR:4.58;95% CI:2.17-9.66)、母又は母方祖母に家族歴有(調整 OR:3.36;95% CI:1.40-8.07)との関連を認めた。外反母趾の重症度が上がると、母趾痛を訴える学生の割合が有意に増加した。

【結論】約 3 割の女子大学生に 1 足ないし両足の外反母趾を認めた。外反母趾の家族歴や母趾痛のある若年女性は外反母趾の可能性があり注意が必要である。

キーワード:外反母趾、女子大生、母趾痛、家族歴、横断調査
